# Artificial Intelligence-Aided Mapping of the Structure–Composition–Conductivity Relationships of Glass–Ceramic Lithium Thiophosphate Electrolytes

**DOI:** 10.1021/acs.chemmater.2c00267

**Published:** 2022-07-20

**Authors:** Haoyue Guo, Qian Wang, Alexander Urban, Nongnuch Artrith

**Affiliations:** † Department of Chemical Engineering, Columbia University, New York, New York 10027, United States; ‡ Columbia Center for Computational Electrochemistry, Columbia University, New York, New York 10027, United States; § Columbia Electrochemical Energy Center, Columbia University, New York, New York 10027, United States; ∥ Materials Chemistry and Catalysis, Debye Institute for Nanomaterials Science, Utrecht University, 3584 CG Utrecht, The Netherlands

## Abstract

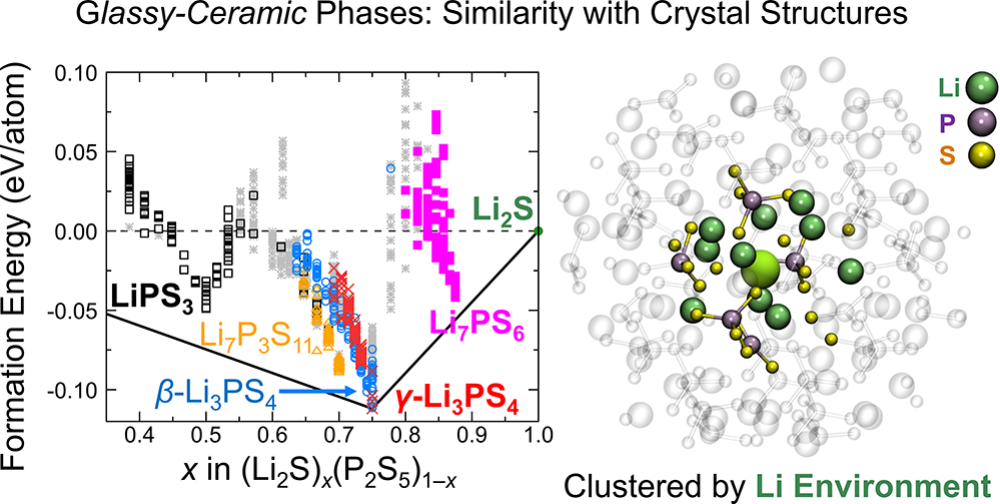

Lithium thiophosphates (LPSs) with the composition (Li_2_S)_*x*_(P_2_S_5_)_1–*x*_ are among the most promising prospective electrolyte materials for solid-state batteries (SSBs), owing to their superionic conductivity at room temperature (>10^–3^ S cm^–1^), soft mechanical properties, and low grain boundary resistance. Several glass–ceramic (*gc*) LPSs with different compositions and good Li conductivity have been previously reported, but the relationship among composition, atomic structure, stability, and Li conductivity remains unclear due to the challenges in characterizing noncrystalline phases in experiments or simulations. Here, we mapped the LPS phase diagram by combining first-principles and artificial intelligence (AI) methods, integrating density functional theory, artificial neural network potentials, genetic-algorithm sampling, and *ab initio* molecular dynamics simulations. By means of an unsupervised structure-similarity analysis, the glassy/ceramic phases were correlated with the local structural motifs in the known LPS crystal structures, showing that the energetically most favorable Li environment varies with the composition. Based on the discovered trends in the LPS phase diagram, we propose a candidate solid-state electrolyte composition, (Li_2_S)_*x*_(P_2_S_5_)_1–*x*_ (*x* ∼ 0.725), that exhibits high ionic conductivity (>10^–2^ S cm^–1^) in our simulations, thereby demonstrating a general design strategy for amorphous or glassy/ceramic solid electrolytes with enhanced conductivity and stability.

## Introduction

**Figure 1:**
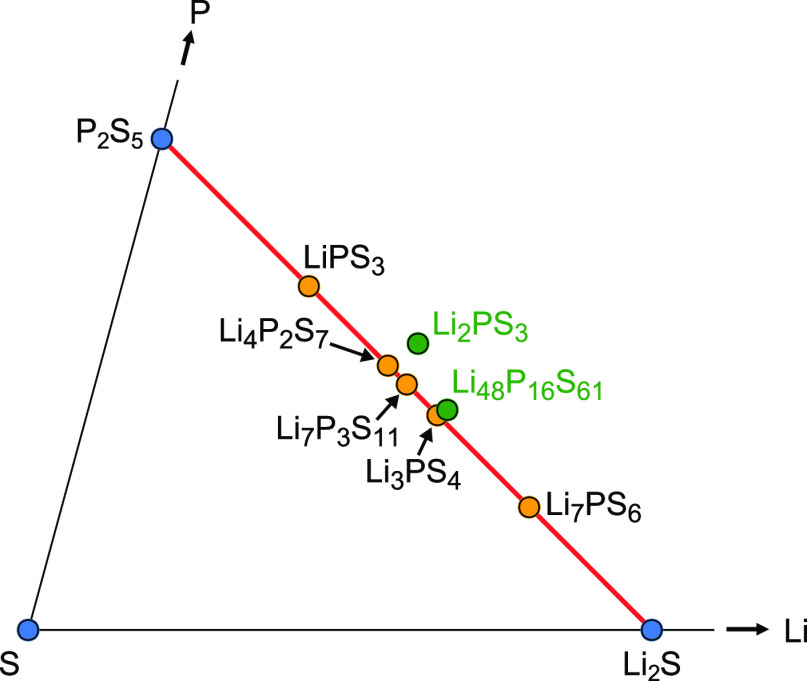
Excerpt from the ternary Li–P–S phase diagram showing reported LPS compositions on and near the Li_2_S–P_2_S_5_ composition line. The materials falling on the right of the red line are sulfur-deficient compositions (green circles).

Solid-state batteries (SSBs) are a prospective alternative to conventional Li-ion batteries (LIBs), in which the flammable liquid electrolytes are replaced with safer solid Li-ion conductors. Additionally, SSBs can potentially enable the use of Li metal anodes and thus significantly higher energy densities.^1−3^ Different classes of materials have been investigated as solid electrolytes (SEs), including oxides, polymers, phosphates, and thiophosphates.^4−7^ Among all the prospective SE materials, lithium thiophosphates (LPSs) with the composition (Li_2_S)_*x*_(P_2_S_5_)_1–*x*_ are among the most promising, owing to their superionic conductivity at room temperature (>10^–3^ S cm^–1^), soft mechanical properties, and low grain boundary resistance.^8,9^ The implementation of LPS glasses as SEs was first reported in 1980,^10^ where it was discovered that the substitution of O with S in phosphates significantly increased the ionic conductivity. In 2006, Mizuno and co-workers observed that the conductivity of LPS materials can be further promoted by partial crystallization of the Li_2_S–P_2_S_5_ glasses.^11,12^ By now, a number of different glass–ceramic (*gc*) LPS compositions have been synthesized and characterized, including LiPS_3_ ((Li_2_S)_0.5_(P_2_S_5_)_0.5_),^13^ Li_2_PS_3_ ((Li_2_S)_0.667_(P_2_S_5_)_0.333_),^14−16^ Li_7_P_3_S_11_ ((Li_2_S)_0.7_(P_2_S_5_)_0.3_),^11,12,17−25^ Li_3_PS_4_ ((Li_2_S)_0.75_(P_2_S_5_)_0.25_),^24,26−34^ and Li_7_PS_6_ ((Li_2_S)_0.875_(P_2_S_5_)_0.125_),^35^ all of which lie on or near the P_2_S_5_–Li_2_S composition line in the Li–P–S phase diagram (Figure 1).

**Figure 2:**
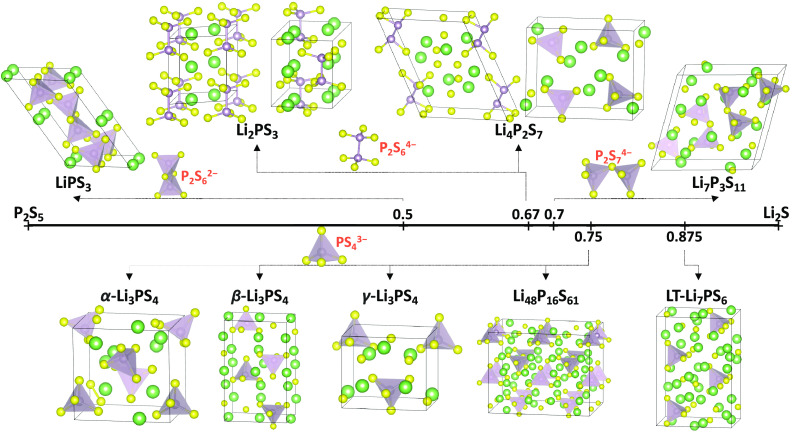
Crystal structures of LPS compositions on and near the Li_2_S–P_2_S_5_ composition line (Li: green; S: yellow; P: purple). The structures are grouped by their local P–S motifs (see Figure 3). Note that Li_2_PS_3_ and Li_48_P_16_S_61_ do not exactly lie on the Li_2_S–P_2_S_5_ composition line, as seen in Figure 1. Note that the structure of the high-temperature α-Li_3_PS_4_ phase has not been fully resolved in the experiment, and our assignment here is speculative.

LPS compositions crystallize in several different crystal structures (Figure 2) that have been extensively characterized with experimental techniques, such as X-ray powder diffraction (XRD) and nuclear magnetic resonance (NMR)^12,13,15,16,24,35−37^ spectroscopy as well as with computational methods.^38−41^ Nevertheless, glass–ceramic (*gc*) LPS-based SEs exhibit both crystalline and noncrystalline phases, and the ionic conductivity of such *gc*-LPS materials is significantly influenced by the glassy phases.^41,42^ Although the crystal structures and electronic properties of LPS have been thoroughly studied, the relationship between structures and Li conductivity in the *gc*-LPS materials has not been well understood, also due to the limitations of experimental and computational techniques for characterizing noncrystalline phases.

**Figure 3:**
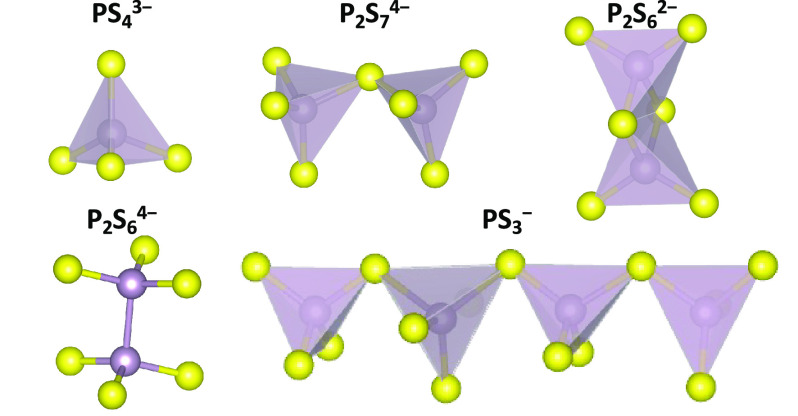
P–S anion motifs in different *gc*-LPSs: ortho-thiophosphate (PS_4_^3–^), pyro-thiophosphate (P_2_S_7_^4–^), hypo-thiodiphosphate (P_2_S_6_^4–^), meta-thiodiphosphate (P_2_S_6_^2–^), and meta-thiophosphate (PS_3_^–^) (S: yellow; P: purple).

In contrast to crystal structures, glasses lack long-range atomic ordering. It has previously been reported that the energy landscape for ion migration can be impacted by subtle variations in the local structures of LPS,^31,41,43,44^ where different local P–S motifs are present depending on the LPS composition. Figure 3 illustrates the five P_*x*_S_*y*_^*n*–^ anionic species commonly observed: ortho-thiophosphate (PS_4_^3–^), pyro-thiophosphate (P_2_S_7_^4–^), hypo-thiodiphosphate (P_2_S_6_^4–^), meta-thiodiphosphate (P_2_S_6_^2–^), and meta-thiophosphate (PS_3_^–^).^45^ Polymeric chains of PS_3_^–^ are only observed in the LPS glasses with low Li_2_S contents (*x* ≤ 0.5 in Figure 2).^45^ Glass–ceramics, containing both crystalline and glassy domains, can be synthesized via ball-milling of the crystalline LPS compounds or by nucleating crystallites in glassy materials via heat treatment.^45−47^ Although the preparation methods can be dramatically different, the relative ratios of local motifs have been found to be similar as long as the composition remains the same.^45^

Different local P–S motifs can affect the Li sites and therefore change the Li ionic conductivity.^41,43,44^ For example, three Li_3_PS_4_ polymorphs, α-, β-, and γ-Li_3_PS_4_, have been synthesized and characterized.^30−32^ α-Li_3_PS_4_ was formed at high temperatures above 746 K,^31^ while β-Li_3_PS_4_ was first obtained at 573 K^30^ and subsequently also at room temperature with other preparation methods.^32^ γ-Li_3_PS_4_ was obtained only at room temperature.^30^ Although the local P–S motifs in the three Li_3_PS_4_ polymorphs are exclusively isolated PS_4_^3–^ tetrahedra, the phases exhibit different cation arrangements and differ in the orientation of the PS_4_^3–^ tetrahedra. Recent theoretical studies proposed that the Li mobility in the β phase is increased because of a paddle-wheel mechanism for Li migration that is observed in β-Li_3_PS_4_ but not in γ-Li_3_PS_4_.^41,43,44^

Previous computational studies mainly focused on the crystalline LPS phases, such as Li_2_PS_3_,^48−50^ Li_7_P_3_S_11_,^21,51−56^ Li_3_PS_4_,^41,43,44,57^ and Li_7_PS_6_.^58^ In some studies, glassy LPS phases were approximated with moderately sized defect structures or molecular dynamics simulations at high temperatures.^41,54,57,59−63^ The impact of local structure motifs on ionic conductivity in *gc*-LPS has recently been investigated by Sadowski and Albe,^64^ who report that the connectivity of PS_*x*_ structural units does not significantly affect the Li conductivity of the glassy phases but that instead the nature of the Li sites is the most important structural factor. However, the Li migration mechanism remains controversial in the literature, since crystalline Li_7_P_3_S_11_ exhibits the highest ionic conductivity despite exhibiting corner-shared PS_4_^3–^ tetrahedra as local P–S motifs.^21,51−55^ In an earlier kinetic study combining reverse Monte Carlo (RMC) modeling and neutron diffraction, it was proposed that the corner-sharing P_2_S_7_^4–^ shields the positive charge of P due to electron transfer between P and bridging S, therefore suppressing Li conduction.^65−67^ However, a later *ab initio* molecular dynamics (AIMD) study found that the flexibility of P_2_S_7_^4–^ ditetrahedra facilitates Li^+^ diffusion.^21^

In essence, only few theoretical studies of amorphous/glassy LPS structures have been reported, and the effect of amorphization on Li conduction has not yet been well understood. Conventional density functional theory (DFT) based AIMD simulations alone are limited to relatively small structure models with ∼200 atoms, which makes it challenging to investigate amorphous phases without long-range ordering. In addition, sampling amorphous phases with such moderately sized structure models using AIMD simulations already required significant computational resources. On the other hand, machine learning potentials trained on first-principles reference data can be efficient and accurate for describing amorphous phases with reasonable computation cost.^68−71^

**Figure 4:**
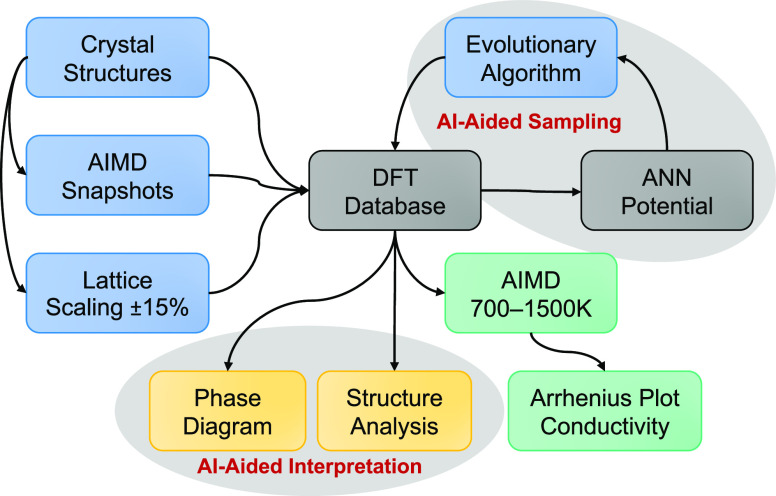
Workflow that was used for the AI-aided mapping of the glass–ceramic (*gc*)-LPS phase diagram by combining density-functional theory (DFT) calculations and accelerated sampling with artificial neural network (ANN) potentials and an evolutionary (genetic) algorithm. All final reported results were obtained from either static DFT calculations (yellow boxes) or DFT-based *ab initio* molecular dynamics (AIMD) simulations (green boxes).

To determine the local atomic structures of *gc*-LPS with varying composition, we mapped the *gc*-LPS phase diagram by integrating DFT,^72^ artificial neural network (ANN) potentials,^73^ evolutionary/genetic-algorithm (GA) sampling, and AIMD simulations as illustrated by the workflow diagram in Figure 4. By varying the compositions along the Li_2_S–P_2_S_5_ composition line using an (artificial intelligence) AI-aided sampling approach, the phase diagram of *gc*-LPS was completed. For each LPS composition, GA global structure optimizations with an ANN potential were performed to determine low-energy atomic configurations. The relevant near-ground-state structures determined by this sampling approach were recomputed with DFT, and all reported final results are based on DFT. The thermodynamic stability and ionic conductivity of glassy/ceramic phases was correlated with local structural motifs by determining similarities of Li sites in glassy and crystalline LPS structures motivated by the recent findings by Sadowski and Albe,^64^ which allowed identifying structure–composition–conductivity relationships. With machine learning accelerated sampling and AIMD simulations, a candidate solid-state electrolyte composition, (Li_2_S)_*x*_(P_2_S_5_)_1–*x*_ (*x* = 0.724), with high ionic conductivity (>10^–2^ S cm^–1^) was identified, which points toward a design strategy for LPS-based SE materials with enhanced conductivity and stability.

## Methods

### Density Functional Theory Calculations

All DFT calculations were carried out with the projector-augmented-wave (PAW) method^74,75^ and the Perdew–Burke–Ernzerhof (PBE) exchange-correlation functional^76^ as implemented in the Vienna *Ab Initio* Simulation Package (VASP)^72,74^ and an energy cutoff of 520 eV. Gaussian smearing with a width of 0.05 eV was used, and total energies were generally converged better than 10^–5^ eV/atom; the final force on each atom was less than 0.02 eV/Å. The first Brillouin zone was sampled using VASP’s fully automatic k-point scheme with length parameter *R*_*k*_ = 25 Å.

Amorphous structure models were generated with AIMD simulations of supercells containing 80–128 atoms. In AIMD simulations, a Gamma k-point scheme was employed to reduce the computational cost. The time step for the integration of the equations of motion was set to 1 fs, and the temperature of the system was set to 1200 K using a Nosé–Hoover thermostat.^77^ To obtain near-ground-state structures as reference for the machine-learning potential (see below), 150 evenly spaced snapshots were extracted from the AIMD trajectories that were reoptimized with DFT at zero Kelvin via geometry optimizations as described above.

To determine ionic conductivities, ∼300 ps long AIMD simulations were performed for select compositions (detailed in the Results section) after at least 50 ps of equilibration at the temperatures 700, 900, 1200, and 1500 K. The ionic conductivities at room temperature and the activation energies were obtained from Arrhenius extrapolation.^69^

### Representation of Atomic Environments

To be suitable as inputs for our machine-learning models, local atomic environments, including atomic positions and species, need to be featurized, i.e., transformed to a vector representation with constant dimension.^78^ In the present work, these feature vectors were derived from the expansion of the radial and angular atomic distribution functions in an orthogonal basis set as described previously.^78^ The expansion of the radial distribution function (RDF) centered on atom *i* is approximated as
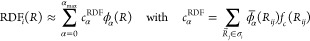
where α = 0, ..., α_max_ is the expansion order, *ϕ*_*α*_ and *ϕ̅*_*α*_ are the basis function corresponding to order α as well as its orthogonal dual function, and *c*_α_^RDF^ are the expansion coefficients. The second sum in the expression of the coefficients runs over the Cartesian coordinates *R⃗*_*j*_ of all atoms *j* within the local environment of atom *i*, *σ*_*i*_, the distance between atom *i* and its neighbor *j* is denoted *R*_*ij*_, and *f*_*c*_ is a cosine cutoff function that smoothly goes to zero at a defined maximal interaction distance. The expansion of the angular distribution function (ADF) is equivalent and yields the expansion coefficients {*c*_α_^ADF^}.

The RDF and ADF expansion coefficients {*c*_α_^RDF^} and {*c*_α_^ADF^} are invariant with respect to the rotation and translation of the atomic structure and the permutation of equivalent atoms, which makes them suitable features of the local structure. To incorporate information about the chemical species within the local atomic environment *σ*_*i*_, the contribution of each atom *j* is weighted with an element-specific weight *w*_*t*_*j*__ (*t*_*j*_ is the type of atom *j*), yielding a second set of expansion coefficients {*c̃*_α_^RDF^} and {*c̃*_α_^ADF^}. The complete feature vector of the local atomic environment of atom *i* is then given by the concatenation of the four sets of expansion coefficients
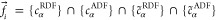
Here, we employed a Chebyshev basis set with a cutoff of 6.0 Å for the radial expansion (expansion order 18) and a cutoff of 3.0 Å for the angular expansion (expansion order 4).^79^ Hence, the dimension of the Chebyshev feature vectors *f⃗*_*i*_ is 2 × (19 + 5) = 48, including also the coefficients for expansion order 0. We used *w*_*t*_*j*__ = −1, 0, +1 to weight the contributions of the three species.

### Machine-Learning Potentials

All machine-learning potential (MLP) simulations were performed with artificial neural network (ANN) potentials^79,80^ as implemented in the atomic energy network package (ænet).^73,79,81^ ANN potentials represent the total energy *E*_tot_ of an atomic structure as the sum of atomic energies, *E*_tot_ = ∑_*i*_^*N*_atom_^*E*_*i*_, where the atomic energies *E*_*i*_ are predicted by ANNs for a given local atomic environment and *N*_atom_ is the number of atoms in the structure. Local atomic environments were represented as described above. An ANN architecture with two hidden layers of 15 nodes each and hyperbolic tangent activation functions was employed. The Broyden–Fletcher–Goldfarb–Shanno (BFGS) method^82^ was employed for the weight optimization. A total of 10% of the reference data were randomly selected as an independent validation set for cross-validation and were not used during training. The training was repeated 10 times for 500 training iterations using different randomly initialized weight parameters, and the ANN potential with the lowest validation-set error was selected.

For accelerated sampling, a specialized ANN potential was trained on a data set containing ∼6000 atomic structures that were generated with the following iterative approach: (i) An initial ANN potential was trained on the LPS crystal structures with lattice parameters scaled between ±15% and randomly perturbed atomic positions from short AIMS simulations at 1200 K; (ii) a number of *gc*-LPS structure configurations were generated with the genetic algorithm sampling approach described below using the ANN potential; and (iii) the 10 structures with lowest ANN potential energy among those sampled were reoptimized using DFT and added to the reference data set. The final ANN potential yields a root-mean-squared error of 1.4 meV/atom and a mean absolute error of 0.6 meV/atom relative to the DFT reference energies in an independent validation set that was not used for training and contained 10% of the structures in our database. As previously demonstrated for amorphous LiSi alloys and LiPON solid electrolytes,^68−70^ specialized ANN potentials constructed based on moderately sized reference data sets can be used in conjunction with DFT for accelerated sampling of amorphous phases.

### Genetic Algorithm Sampling

With the specialized ANN potential, the amorphous phases along the Li_2_S–P_2_S_5_ composition line were sampled with a genetic-algorithm (GA) as implemented in the atomistic evolution (ævo) package (http://ga.atomistic.net),^68^ following previously reported strategies that are briefly described in the following.^68−70^ Although glassy phases lack long-range ordering, it can be expected that the local atomic motifs in *gc*-LPS phases resemble those of the known LPS crystalline phases (Figure 3). The phase diagram of LPS compositions was therefore constructed by varying the stoichiometry *x* in (Li_2_S)_*x*_(P_2_S_5_)_1–*x*_ via removing Li_2_S or P_2_S_5_, respectively, from supercells of the known LPS crystal structures. The approach is as follows:

1. A supercell of one of the crystal structures LiPS_3_, Li_7_P_3_S_11_, β-Li_3_PS_4_, γ-Li_3_PS_4_, or Li_7_PS_6_ is chosen as the *parent structure*;
2. The GA is used to identify combinations of 2 Li and 1 S atoms that can be removed with low formation energy relative to Li_2_S and P_2_S_5_;
3. The created Li_2_S deficient composition is optimized with DFT; and
4. The optimized structure is taken to be the new parent structure, and the algorithm continues with step (2).

We used the same technique to sample in the opposite direction on the Li_2_S–P_2_S_5_ composition line by removing 2 P and 5 S atoms at each step (instead of 2 Li and 1 S atoms).

The GA employed a population size of 32 trials and a mutation rate of 10%. For each composition, at least 10 lowest energy structure models identified with the ANN-GA approach were selected and fully relaxed with DFT to obtain the first-principles phase diagram. We emphasize that the GA sampling approach yields, by design, DFT optimized structures and their DFT energies.

### Formation Energy

For any given structure and composition (Li_2_S)_*x*_(P_2_S_5_)_1*–x*_ the corresponding formation energy per atom was calculated as
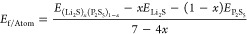
1where *E* is the total energy of a specific configuration as predicted by DFT; *x* is the molar fraction of Li_2_S in the LPS composition; and *E*_Li_2_S_ and *E*_P_2_S_5__ are constant and are equal to the total energy per formula unit of bulk Li_2_S and P_2_S_5_, respectively. For any given composition, the configuration with a lower formation energy is thermodynamically favored at zero Kelvin. The stabilities of different compositions can be compared by constructing the lower convex hull of the formation energies to obtain the phase diagram.^68^

### Structure Similarity and Classification

Low-energy amorphous LPS structures were compared with the known LPS crystal structures by their connectivity of PS_4_ tetrahedra, following a previous study.^69^ In addition, we analyzed structure similarities based on structure *fingerprints*, i.e., each considered structure was transformed to a feature vector with constant dimension. These structure fingerprints were constructed based on the Chebyshev descriptors of local atomic environments, mentioned above in the context of ANN potentials.^79^ The local environment of an atom *i* is represented by a Chebyshev feature vector *f⃗*_*i*_. To construct a structure fingerprint *F⃗*, the first *K* moments of the distribution of the atomic feature vectors were calculated, where the *k*th moment is given by
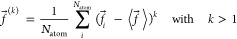
2and ⟨*f⃗*⟩ *=f⃗*^(1)^ is the mean atomic feature vector (the first moment). The structure fingerprint is then the union (i.e., vector concatenation) of the distribution moments, *F⃗* = *f⃗*^(1)^ ∪ *f⃗*^(2)^ ∪ ..., until a maximum moment. In practice, we found that truncating after the second moment already yielded unique structure fingerprints that can distinguish all atomic structures in our database. Atom-type specific structure fingerprints can be constructed by including only atomic feature vectors for the local atomic environments of select atomic species. We made use of this approach by constructing structure fingerprints based on only the local atomic environment of Li atoms. Finally, we reduced the dimension of the structure fingerprints by performing a principal component analysis (PCA) after data standardization, using the PCA and StandardScaler implementations of the *scikit-learn* library.^83^ We found 10 principal components to be sufficient, which can explain 85% of the data variance. Hence, each atomic structure in our database could be uniquely represented by a fingerprint vector with 10 components.

Using the structure fingerprints, we define the similarity *S*_p_ of two atomic structures as the Pearson correlation coefficient
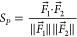
3where *F⃗*_1_ and *F⃗*_2_ are two (dimension-reduced) structure fingerprints. Furthermore, we performed a cluster analysis of the structure fingerprints using the *k*-means approach as also implemented in *scikit-learn*.^83^

## Results

### Phase Diagram along the Li_2_S–P_2_S_5_ Composition Line

**Figure 5:**
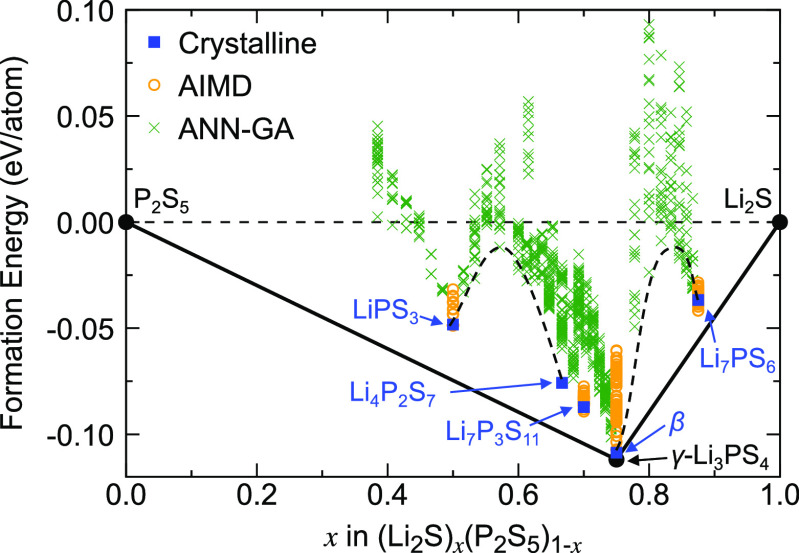
Computational LPS phase diagram along the P_2_S_5_–Li_2_S composition line. Only the γ-Li_3_PS_4_ phase lies on the lower convex hull (black solid line) and is thus predicted to be thermodynamically stable at zero Kelvin. Metastable crystalline phases are indicated by blue squares, and structures generated from *ab initio* molecular dynamic (AIMD) simulations and genetic-algorithm (GA) sampling with the ANN potential are shown as orange circles and green crosses, respectively. Two miscibility gaps are indicated with dashed black lines to guide the eye.

Our computational sampling of the Li_2_S–P_2_S_5_ composition line started with 13 LPS crystal structures with the formula units LiPS_3_,^13^ Li_2_PS_3_,^14−16^ Li_4_P_2_S_7_,^58,60^ Li_7_P_3_S_11_,^17^ α-Li_3_PS_4_,^31^ β-Li_3_PS_4_,^30,32^ γ-Li_3_PS_4_,^30^ Li_48_P_16_S_61_,^84^ and low-temperature (LT)-Li_7_PS_6_^35^ that had previously been reported based on experimental characterization and/or theoretical modeling. The crystal structures, which were obtained from the Inorganic Crystal Structure Database (ICSD)^85^ and the Materials Project (MP)^86^ database, are shown in Figure 2. The DFT formation energies of the crystalline LPS phases relative to Li_2_S and P_2_S_5_, the end points of the composition line, are shown in Figure 5. As seen in this phase diagram, only one crystal structure (γ-Li_3_PS_4_) appears on the lower convex hull of the formation energies and is thus predicted to be thermodynamically stable at zero Kelvin. The previously reported superionic conductors, β-Li_3_PS_4_^30,32^ and Li_7_P_3_S_11_,^17^ are 3.2 meV/atom and 17.2 meV/atom above the convex hull, indicating that they are metastable at zero Kelvin. However, the energy difference between β-Li_3_PS_4_ and γ-Li_3_PS_4_ is small (3.2 meV/atom) compared to the thermal energy per degree of freedom at room temperature (∼26 meV), so that it is plausible that the β polymorph can be thermodynamically stable at room temperature. Note that the crystal structure of Li_4_P_2_S_7_^58,60^ is a theoretical prediction from the literature and has not been characterized experimentally yet, which is consistent with its comparatively high decomposition energy of 23.5 meV/atom in our phase diagram.

Also shown in the phase diagram of Figure 5 are structures that were generated using the ANN-GA sampling methodology described in the Methods section by removing Li_2_S or P_2_S_5_ from supercells of the crystal structures. This composition sampling yielded low-energy structures with structural disorder and no symmetry, as one would expect for *amorphous* or *glassy* phases, while still exhibiting local similarities with the parent crystal structures from which they were derived. At zero Kelvin, these glass–ceramic structures are also predicted to be thermodynamically unstable, though they might be stabilized at synthesis temperatures due to their high entropy (entropy control) or via kinetic trapping.

As seen in the phase diagram, the ANN-GA sampling identified two miscibility gaps between LiPS_3_ and Li_4_P_2_S_7_ and between Li_3_PS_4_ and Li_7_PS_6_, respectively. This means that compositions (Li_2_S)_*x*_(P_2_S_5_)_1*–x*_ with 0.5 < *x* < 0.667 and 0.75 < *x* < 0.875 will likely phase separate instead of forming a solid solution, in agreement with previous experimental observations (see also the Discussion section).^24^ However, between Li_4_P_2_S_7_ and Li_3_PS_4_, amorphous structures with low energies above the convex hull (<90 meV/atom) were found. It can, therefore, be expected that compositions with 0.667 < *x* < 0.75 can be more readily synthesized.

### Structural Motifs of the Sampled LPS Phases

The LPS crystal structures shown in Figure 2 are composed of a variety of local motifs (Figure 3), which have previously been found to affect the ionic conductivity and the Li transport mechanisms.^45^ Isolated PS_4_^3–^ tetrahedra are mostly observed in the *gc*-LPS compositions with high Li_2_S content (*x* ≥ 0.75), such as α-Li_3_PS_4_, β-Li_3_PS_4_,^30,32^ γ-Li_3_PS_4_,^30^ and Li_7_PS_6_.^35^ The P_2_S_7_^4–^ motif, consisting of two corner-sharing PS_4_ tetrahedra, is the main building block of the Li_7_P_3_S_11_ crystal structure^17^ as well as glassy LPS compositions with *x* < 0.75. The P_2_S_6_^2–^ motif, formed by two edge-sharing PS_4_ tetrahedra, is observed in *gc*-LPS with *x* ≤ 0.6 and is the only local motif in LiPS_3_ crystals.^13^ The P_2_S_6_^4–^ with direct P–P bonding is typically present in *gc*-LPS with 0.6 ≤ *x* ≤ 0.7.^24^ Note that the oxidation state of P is +4 only in the P_2_S_6_^4–^ motif, while it is +5 in all other local motifs. The P_2_S_6_^4–^ motif also occurs in Li_2_PS_3_,^14−16^ which is a sulfur-deficient composition that is not on the Li_2_S–P_2_S_5_ composition line.

**Figure 6:**
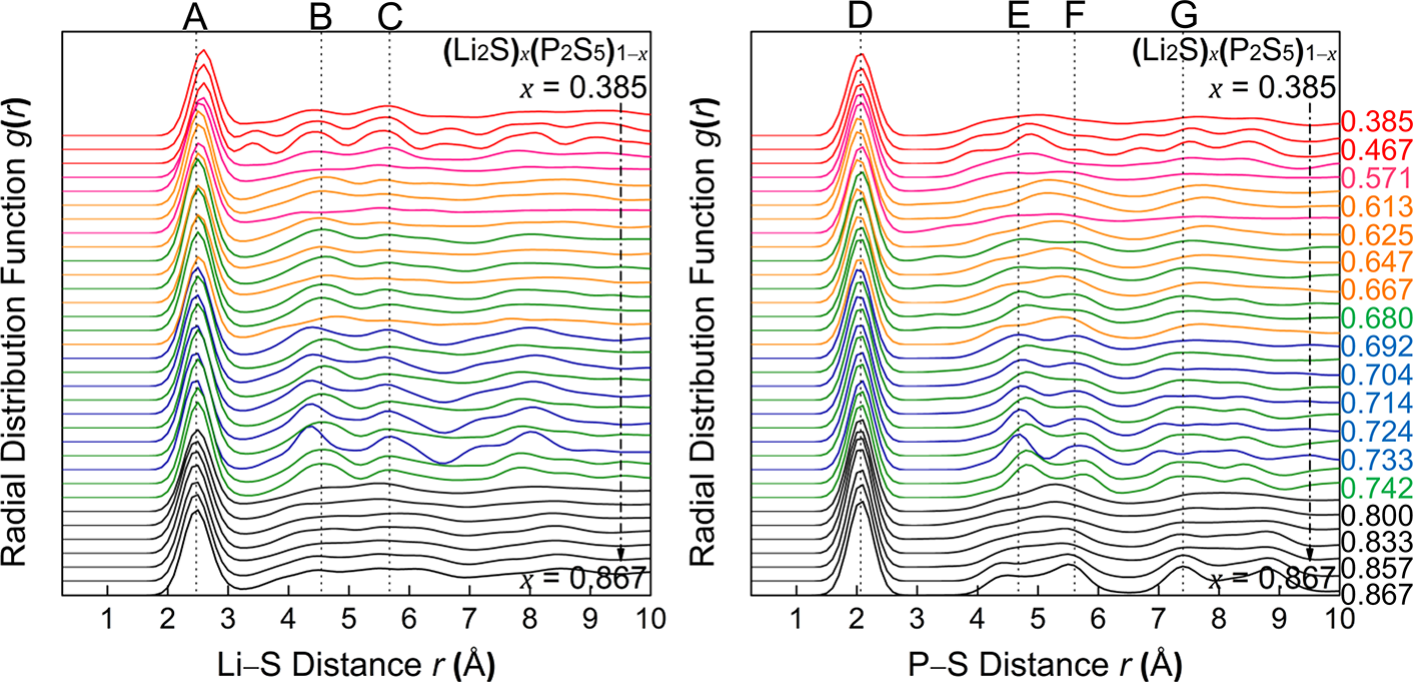
Calculated Li–S (left) and P–S (right) radial distribution functions (RDF) of glass–ceramic (*gc*) (Li_2_S)_*x*_(P_2_S_5_)_1–*x*_ (*gc*-LPS) phases with varying compositions from *x* = 0.385 to *x* = 0.867 (the composition of every other line is labeled on the right). Each line is an average RDF of the 10 lowest-energy structures at a specific composition. The *gc*-LPS structures were generated by genetic-algorithm modification of a parent structure (see Methods section), and the color represents the parent crystal structure (i.e., black: Li_7_PS_6_, blue: γ-Li_3_PS_4_, green: β-Li_3_PS_4_, orange: Li_7_P_3_S_11_, pink and red: LiPS_3_). The dashed lines indicate measured RDFs from experiments: Peak A,^41,42,62^ B,^24,41,42^ C,^24,41^ D,^15,24,41,62^ E,^15,24,41^ F,^24,41^ and G.^24^

To better understand the local structures of the ANN-GA sampled *gc*-LPS phases, we computed the radial pair distribution functions (RDFs) for P–S and Li–S in *gc*-LPS compositions with 0.385 ≤ *x* ≤ 0.867 as shown in Figure 6 and Figure S1. As seen in the figures, and as expected, the RDFs of the generated *gc*-LPS structures exhibit features of the crystal structure RDFs but show broadened peaks with shifted peak positions. In general, with decreasing amount of Li_2_S in *gc*-LPS, the main Li–S peak shifts to greater distances, which is caused by the formation of corner-sharing motifs, in agreement with previous reports.^42,60,61^ Note that for a large fraction of the *gc*-LPS structures (∼1/3) the shape of the RDF differs significantly from that of the parent structure; i.e., the RDFs of derived structures exhibit different peaks than the RDF of the parent crystal structure. Instead, structures with the same composition that were derived from two different parent structures exhibit similar peaks, indicating that these compositions have a strong preference for specific structural motifs. This is especially evident in the S–S RDF shown in Figure S1 and indicates that *gc*-LPS with compositions in between the crystalline phases may exhibit multiple different local structural motifs found in the neighboring (by composition) crystalline LPS.

**Figure 7:**
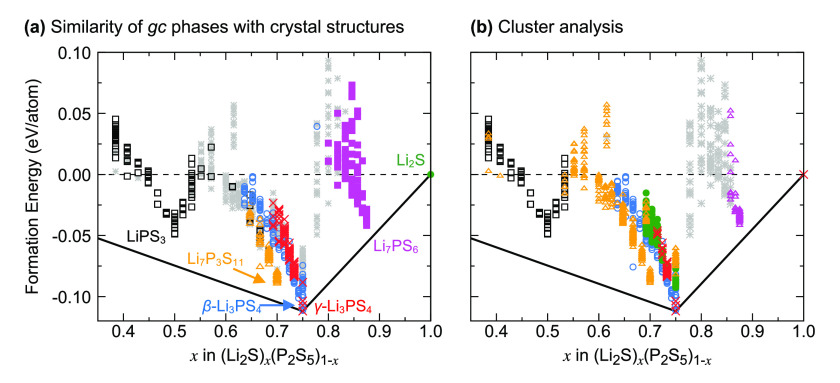
Analysis of the local atomic Li environment in the simulated glass–ceramic (*gc*) phases. (a) The symbols and color coding indicate the crystal structure that is most similar based on the Pearson correlation of the structural fingerprints. Structures that are not strongly correlated with any crystal structure are shown as gray stars. (b) Grouping of similar structures with *k*-means clustering of the Li local atomic environments. The structures within the same cluster are shown with the same symbol and color.

As discussed in the Introduction section, the P–S structural building blocks alone cannot explain all the differences in the Li conductivities, and RDFs capture only one specific structural feature, namely, radial correlations. The structural fingerprints introduced in the Methods section are more general. Figure 7 shows an analysis of the structural fingerprints of all structures in our database to identify and visualize similarities more directly. For this comparison, each structure was represented by a structure fingerprint based on the local atomic environments of all Li atoms, which can be assumed to be an important criterion for Li conductivity.

In Figure 7a, the similarities of each structure with the reference crystal structures LiPS_3_, Li_7_P_3_S_11_, β-Li_3_PS_4_, γ-Li_3_PS_4_, Li_7_PS_6_, and Li_2_S are shown. The Pearson correlation *S*_*p*_ of the structure descriptors (see Methods section) was used as a measure of similarity, and structures with *S*_*p*_ < 0.4 for all of the crystal structures were considered not to be similar to any of the reference structures. With this threshold, more than 95% of the structures in our database can be assigned uniquely to a reference crystal structure (see Figure S2). Most of the structures derived from either LiPS_3_ or Li_7_PS_6_ remain similar to their parent structure during sampling, leading to distinct clusters for these structures in Figure 7a. However, trends are more complicated for compositions near Li_3_PS_4_ (*x* = 0.75). Within the narrow composition range 0.70 ≤ *x* ≤ 0.75, the structures closest to the ground-state hull change in character from Li_7_P_3_S_11_ to structures that are similar to β-Li_3_PS_4_ and γ-Li_3_PS_4_.

Instead of classifying the sampled glass–ceramic structures by their similarities to reference crystal structures, Figure 7b shows the result of an unsupervised classification of Li environments using *k*-means clustering. The predicted grouping resembles the one shown in Figure 7a but with clearer trends in phase stabilities. At the composition Li_3_PS_4_, the cluster analysis finds that the Li environment changes with increasing energy, which we can attribute to the γ, β, and α polymorphs. At high energies above the ground state hull, a fourth class of the Li environment is found of which Li_7_P_3_S_11_ is also a member, though it is unlikely that these structures can be synthesized at any conditions.

### Li Conductivity

**Figure 8:**
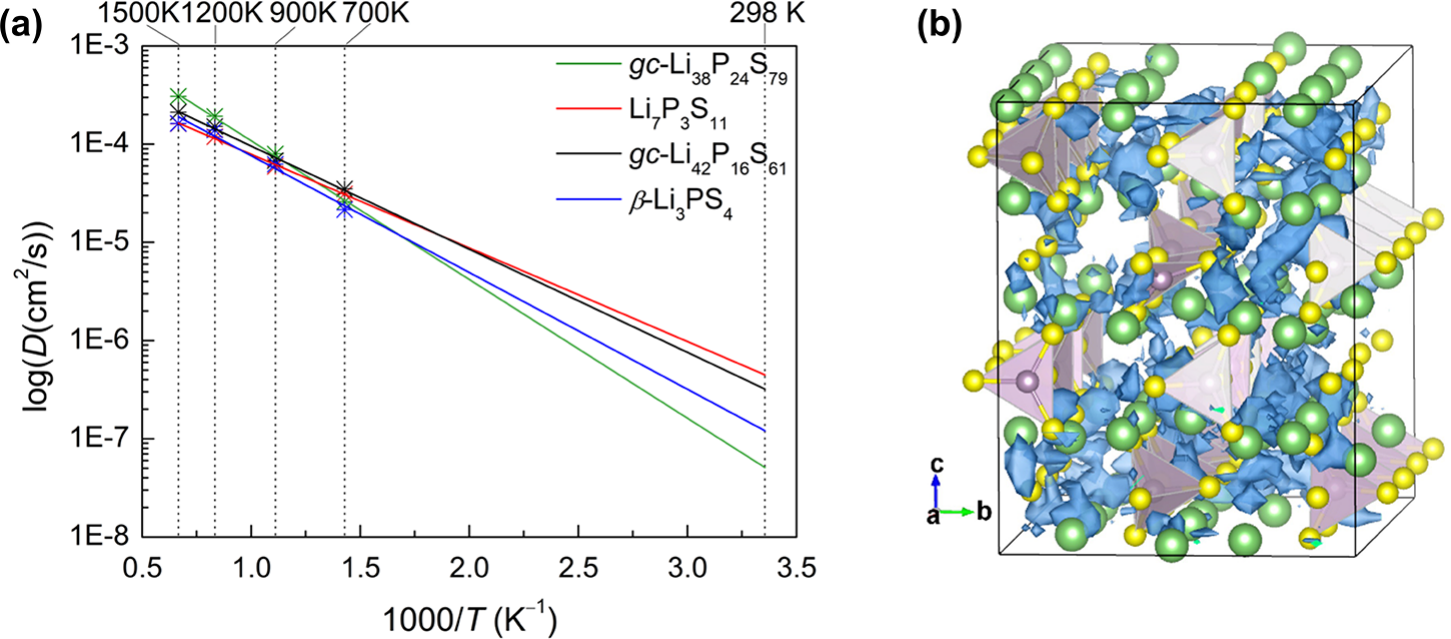
(a) Arrhenius plot of the calculated diffusivities from AIMD simulations at elevated temperatures (700, 900, 1200, and 1500 K) of selected *gc*-LPS compositions (*gc-*Li_38_P_24_S_79_, *gc-*Li_42_P_16_S_61_, Li_7_P_3_S_11_, and β-Li_3_PS_4_) and extrapolation to room temperature. (b) Isosurface of the probability density distribution (blue) *P*(**r**) of Li^+^ ions in *gc-*Li_42_P_16_S_61_ at 700 K (Li: green; S: yellow; P: purple).

The cluster analysis of the Li atom environments discussed in the previous section indicates that the lowest-energy *gc*-LPS phases with compositions between Li_7_P_3_S_11_ (*x* = 0.70) and Li_3_PS_4_ (*x* = 0.75) exhibit the same type of Li environments as the superionic conductor β-Li_3_PS_4_. Given this energetic preference, it is likely that β-Li_3_PS_4_-like Li environments are present in as-synthesized *gc*-LPS within this composition range or would form over time. To determine if this similarity also translates to Li conductivity, we performed AIMD simulations for a glass–ceramic LPS with composition *gc-*Li_42_P_16_S_61_ (*x* = 0.724), the two neighboring crystalline phases (β-Li_3_PS_4_ and Li_7_P_3_S_11_), and a composition outside the target range, *gc-*Li_38_P_24_S_79_ (*x* = 0.613), for comparison. The ionic conductivities at room temperature were obtained from Arrhenius extrapolation (Figure 8a and Figure S3) and are compiled in Table 1. The table also shows measured ionic conductivities in *gc*-LPS from the literature, which are sensitive with respect to the experimental conditions, e.g., temperature and pressure. Samples prepared under different conditions may exhibit different local motifs, leading to a wide range of measured conductivities.^21−23^

**Table 1:** Comparison of Calculated Activation Energy and Li Conductivity of Selected *gc*-LPS Phases (i.e., *gc*-Li_38_P_24_S_79_, *gc-*Li_42_P_16_S_61_, Li_7_P_3_S_11_, and β-Li_3_PS_4_) with Experimental Measurements

			activation energy (eV)			ionic cond. RT (mS cm^–1^)		
*x*	formula	moiety	our AIMD	ref. AIMD	exp.	our AIMD	ref. AIMD	exp.
0.613	*gc*-Li_38_P_24_S_79_	P_2_S_7_^4–^, PS_4_^3–^	0.282	N/A	N/A	3.45	N/A	N/A
0.7	Li_7_P_3_S_11_	P_2_S_7_^4–^, PS_4_^3–^	0.189	0.189^55^	0.187^11^	46.9	57^21^	3.2^11,12^
				0.187^21^	0.124^12^		72.16^54^	4.1^18^
				0.17^54^	0.145^18^			5.2^19^
				0.38^40^	0.176^20^			17^20^
					0.18–0.209^21^			1.3–11.6^21^
					0.29–0.425^22^			0.022–8.6^22^
					0.289–0.401^23^			0.05–4^23^
					0.451^24^			
0.724	*gc*-Li_42_P_16_S_61_	PS_4_^3–^	0.208	N/A	N/A	33.1	N/A	N/A
0.75	β-Li_3_PS_4_	PS_4_^3–^	0.236	0.1, 0.35^61^	0.49^27^	14.3	4.35^57^	0.2^28^
				0.23^40^	0.352^28^		7,^41^ 19^41^	0.16^32^
				0.35^44^	0.16^31^			0.28^24^
				0.22, 0.25^41^	0.356^32^			
					0.399^24^			

As shown in Table 1, our predicted ionic conductivity and activation energy in crystalline Li_7_P_3_S_11_ is in good agreement with previously reported experimental measurements and theoretical calculations. The differences are greater for the β-Li_3_PS_4_ phase, where the agreement with previous simulations is good but predicted conductivities are significantly greater than those observed in experiments. This has to be expected, since the metastable β phase is more challenging to characterize experimentally as well as in simulations. Hence, the data for the β phase is subject to greater uncertainties.

The ionic conductivity of *gc*-Li_42_P_16_S_61_ is high (33.1 mS cm^–1^) and lies between the conductivities of crystalline Li_7_P_3_S_11_, 46.9 mS cm^–1^, and β-Li_3_PS_4_, 14.3 mS cm^–1^. In comparison, the other amorphous phase, *gc*-Li_38_P_24_S_79_ (*x* = 0.613), has a significantly lower ionic conductivity of 3.45 mS cm^–1^ and higher activation energy of 0.282 eV (Table 1), showing that noncrystallinity alone is not responsible for the high conductivity. Note that energetically *gc*-Li_42_P_16_S_61_ is only 28.0 meV/atom above the ground-state hull and is likely synthesizable, whereas *gc*-Li_38_P_24_S_79_ lies in a miscibility gap (70.5 meV/atom above the hull) in the phase diagram (Figure 5) and is highly unstable, so that the composition would likely phase separate on longer time scales.

## Discussion

In the present work, we mapped the phase stability and structure of glass–ceramic lithium thiophosphates along the Li_2_S–P_2_S_5_ composition line. Our calculations identified two miscibility gaps in the composition ranges (Li_2_S)_*x*_(P_2_S_5_)_1–*x*_ with 0.5 ≤ *x* ≤ 0.667 and 0.75 ≤ *x* ≤ 0.875, predicting that solid solutions with such compositions would be challenging to synthesize and likely to phase separate at room temperature. Dietrich et al. previously conducted an experimental study of glass–ceramic LPS compounds with 0.6 ≤ *x* ≤ 0.8 and found that LPS (*x* = 0.8) phase separates into Li_3_PS_4_ (*x* = 0.75) and Li_2_S (*x* = 1.0),^24^ in agreement with our prediction. However, the same authors reported the successful preparation and characterization of LPS (*x* = 0.6), which should also be unstable based on the calculated phase diagram. A possible explanation for this discrepancy could be sulfur deficiency in the compositions, since Li_4_P_2_S_6_ is a known decomposition product of *gc*-Li_4_P_2_S_7_^13^ and an attractor in the phase diagram (see Figure 1). The impact of such off-stoichiometries deserves a more detailed study in the future.

**Figure 9:**
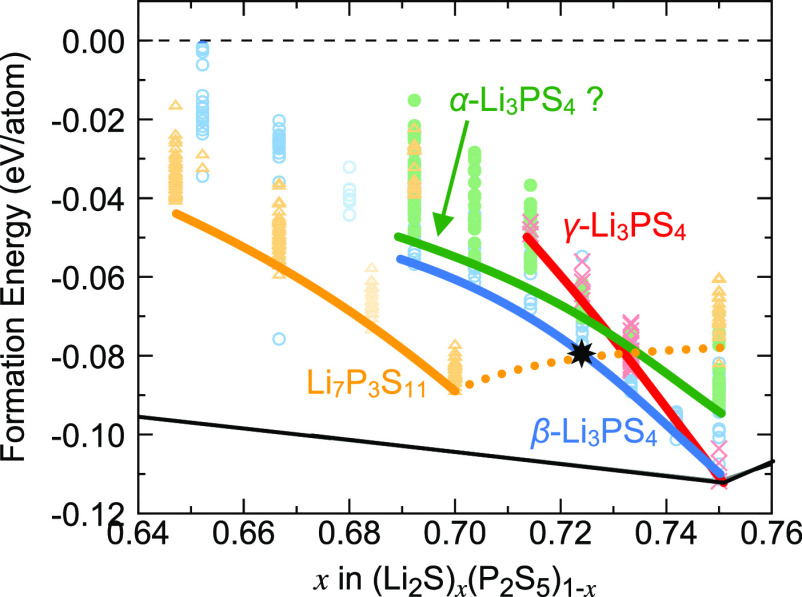
Analysis of the LPS phase diagram near the composition Li_3_PS_4_ = (Li_2_S)_0.75_(P_2_S_5_)_0.25_, based on the cluster analysis of Figure 7. The energetic order of structures with Li environments similar to the β- and γ-Li_3_PS_4_ changes as the Li_2_S content decreases, and structures that are similar to γ-Li_3_PS_4_ are destabilized relative to those similar to β-Li_3_PS_4_. The identified glass–ceramic phase with good Li conductivity, *gc*-Li_42_P_16_S_61_, is indicated by a star. Note that the structure of the high-temperature α-Li_3_PS_4_ phase has not been fully resolved in experiment, and our assignment here is speculative.

The calculated phase diagram shows that the superionic LPS compounds are metastable and therefore prone to decomposition, which is in agreement with previous experimental and computational work discussed in the Introduction section. A particular challenge is that the β-Li_3_PS_4_ polymorph, a superionic Li conductor, is unstable compared to the γ-Li_3_PS_4_ polymorph, which exhibits poor Li conductivity. The cluster analysis of Li environments (Figure 7) points toward an opportunity, since Li environments similar to those in β-Li_3_PS_4_ become stable compared to those of the γ phase when the composition is slightly altered from the ideal Li_3_PS_4_ (*x* = 0.75) to *x* < 0.75. This relative destabilization of the γ phase is visualized in Figure 9. Indeed, our AIMD simulations confirm that the glass–ceramic *gc*-Li_42_P_16_S_61_ (*x* = 0.724) exhibits a high Li conductivity of 33 mS cm^–1^. The RDF analysis of Figure 6 further shows that the P–S and Li–S distribution in *gc*-Li_42_P_16_S_61_ derived from β-Li_3_PS_4_ still resembles that of the parent phase. As seen in Figure 8b, the *gc*-Li_42_P_16_S_61_ structure exhibits both well-ordered and disordered domains, and the Li probability distribution is greater in the ordered regions. This further indicates that reminiscence of the crystalline phase is important for Li conductivity in this *gc*-LPS composition. Though we note that the PS_*x*_ motifs do not generally control the Li environments, there are structures with similar P–S RDFs but different Li environments. An example is analyzed in Supporting Information Figure S4.

Similar to the known crystalline LPS superionic conductors, the here identified LPS composition is also metastable and thermodynamically unstable with respect to decomposition into P_2_S_5_ and Li_3_PS_4_ at zero Kelvin; i.e., it is above the convex hull of formation energies. It has previously been established that knowledge of the energy above the convex hull is insufficient to predict synthesizability^87^ and that the thermodynamic limit for the synthesis of metastable compounds is chemistry-dependent.^88^ On the other hand, a wide range of different *gc*-LPS compositions have previously been reported (e.g., see ref (33)), indicating that glassy–ceramic phases can be synthesized even when their energy is more than the thermal energy at room temperature (26 meV) above the formation energy hull. Unlike crystalline phases, *gc*-LPS phases such as the predicted *gc*-Li_42_P_16_S_61_ benefit from entropy stabilization at finite temperatures. Furthermore, and unlike other glass–ceramic Li conductors, the desired phase with β-Li_3_PS_4_-like Li environments is predicted to be the lowest in energy at the composition (Li_2_S)_*x*_(P_2_S_5_)_1–*x*_ with *x* = 0.724, which means that the phase, if it can be synthesized, could be expected to be shelf-stable at room temperature.

Taken together, the observations made in the present work led to the following design strategy for amorphous solid Li conductors: (1) If Li superionic conductors within a given composition space (such as Li_2_S–P_2_S_5_) are known but are unstable due to phase transitions, the local atomic environment of the Li sites can be taken as a design target, in agreement with previous findings.^64^ (2) Potentially stable superionic conductors can then be identified by searching for regions within the composition space that energetically favor the target Li site environment over other environments.

Finally, we stress that our computational study is subject to approximations, and an experimental confirmation is warranted. The most significant approximation in the present study is the generation and representation of glass–ceramic phases, which was necessarily limited to comparatively small structure sizes and nonexhaustive sampling. Though, based on previous work,^68,69^ ANN-potential accelerated sampling yielded a sufficiently good approximation of the true LPS composition and structure space that the predicted phase diagram and the identified trends in Li environments can be expected to be robust. Another limitation of the present study is that it only considered the Li_2_S–P_2_S_5_ composition line, even though sulfur-deficient LPSs have been reported. The impact of such off-stoichiometries, alluded to in the above discussion, deserves its own investigation.

## Conclusions

We mapped the phase diagram of lithium thiophosphate, (Li_2_S)_*x*_(P_2_S_5_)_1–*x*_, solid electrolytes using first-principles calculations with AI-aided sampling and structure similarity analysis. The phase diagram exhibits two pronounced miscibility gaps, so that compositions with 0.5 < *x* < 0.667 and 0.75 < *x* < 0.875 are prone to phase separation at room temperature even if they can be synthesized. We showed that glassy/ceramic phases with compositions 0.70 < *x* < 0.75 are more likely to be stable because of their lower decomposition energies and exhibit Li sites with local structural environments similar to those in the superionic conductor β-Li_3_PS_4_. This led us to propose a candidate solid-state electrolyte composition, (Li_2_S)_*x*_(P_2_S_5_)_1–*x*_, with *x* = 0.724, that exhibits high ionic conductivity (>10^–2^ S cm^–1^) in simulations, demonstrating a design strategy for glassy or amorphous solid-electrolyte materials with good conductivity and stability.

## Supporting Information Available

A figure with additional radial distribution functions, a figure with additional details of the similarity analysis, mean squared displacement plots, and an analysis of representative *gc*-LPS structures (PDF)

## Notes

This work made use of the free and open-source atomic energy network (ænet) package. The ænet source code can be obtained either from the ænet Web site (http://ann.atomistic.net) or from GitHub (https://github.com/atomisticnet/aenet). The evolutionary algorithm package, ævo, can also be obtained from the GitHub (https://github.com/atomisticnet/aevo). The reference LPS data sets can be obtained from the Materials Cloud repository (10.24435/materialscloud:j5-tz). The data set contains atomic structures and interatomic forces in the XCrySDen structure format (XSF),^89^ and total energies are included as additional meta information.
