# Supplementary material for: Artificial Intelligence-Aided Mapping of the Structure–Composition–Conductivity Relationships of Glass–Ceramic Lithium Thiophosphate Electrolytes
Source: Chem Mater. 2022 Jul 20;34(15):6702–12. doi: 10.1021/acs.chemmater.2c00267 (PMC9367015; doi:10.1021/acs.chemmater.2c00267)
Supplement: Supplementary file 1 — cm2c00267_si_001.pdf [file cm2c00267_si_001.pdf]

Supporting Information

Artificial Intelligence-Aided Mapping of the Structure-Composition-Conductivity Relationships of Glass-Ceramic Lithium Thiophosphate Electrolytes

Haoyue Guo[a], *Qian Wang*[a], *Alexander Urban*[† a,b,c], *and Nongnuch Artrith** [a,b,d]

[a]Department of Chemical Engineering, Columbia University, New York, NY 10027, USA
[b]Columbia Center for Computational Electrochemistry, Columbia University, New York, NY 10027, USA
[c]Columbia Electrochemical Energy Center, Columbia University, New York, NY 10027, USA

[d]Materials Chemistry and Catalysis, Debye Institute for Nanomaterials Science, Utrecht University, 3584 CG Utrecht, The Netherlands

†E-mail: a.urban@columbia.edu *E-mail: n.artrith@uu.nl

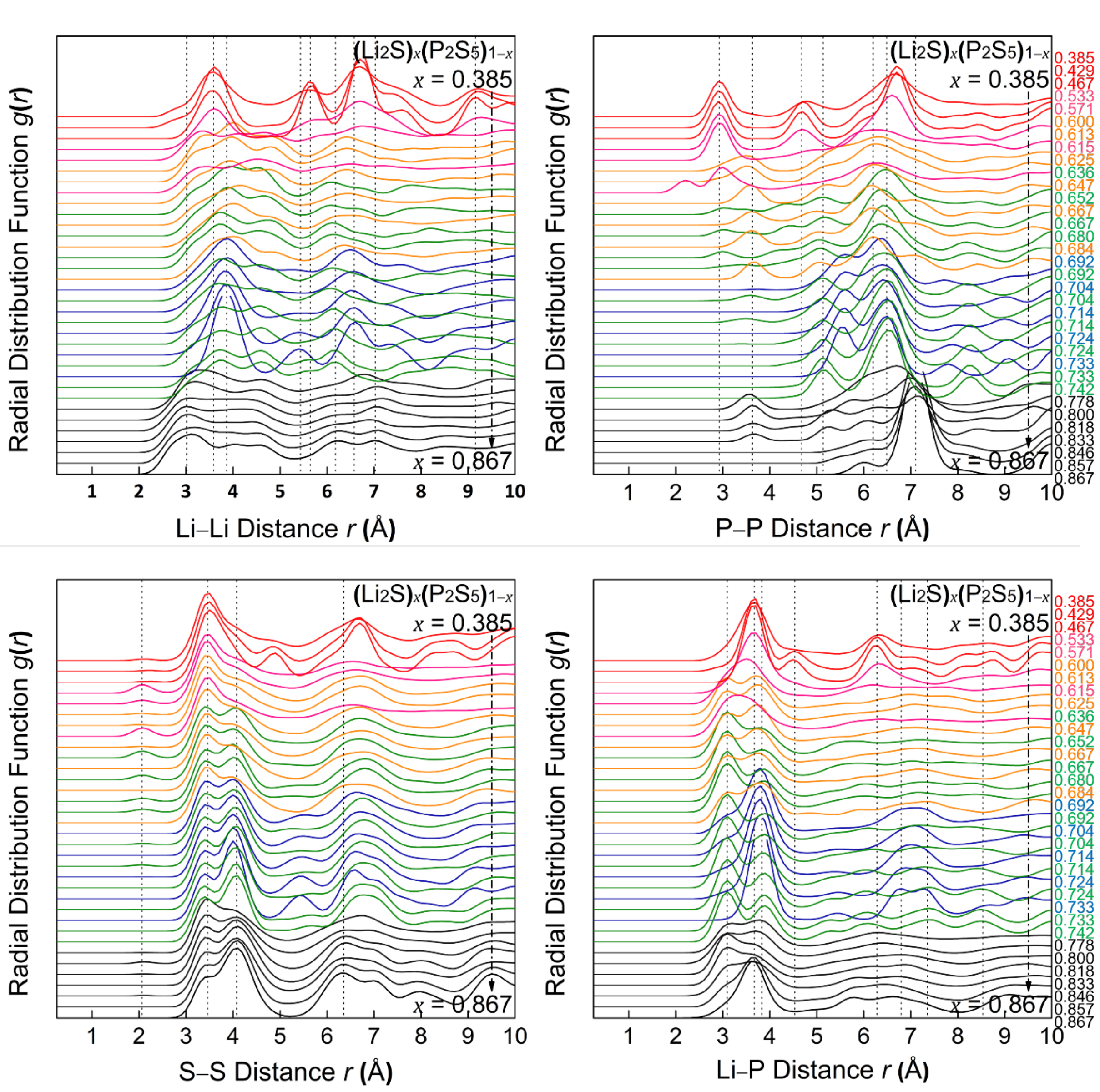

Figure S1. Calculated Li–Li, P–P, S–S and Li–P radial distribution functions (RDF) of glass-ceramic (*gc*) $(Li_2S)_x(P_2S_5)_{1-x}$ (*gc*-LPS) phases with changing compositions from x = 0.867 to x = 0.385. Each line is an average RDF of ten lowest energy structures at certain composition. Since the *gc*-LPS structures are generated with genetic-algorithm (details in Methods Section), the color represents their parent crystalline structure (*i.e*., black: Li_7PS_6, blue: γ-Li_3PS_4, green: β-Li_3PS_4, orange: $Li_7P_3S_{11}$, pink and red: $LiPS_3$). The dashed lines represent measured RDF for crystalline phases.[1–8]

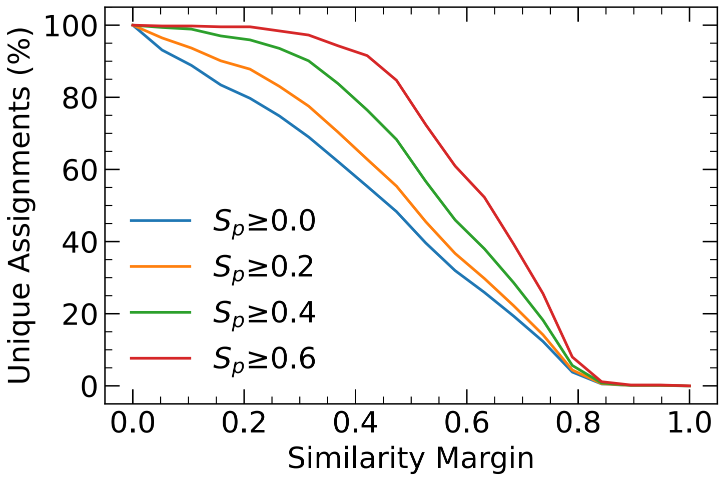

Figure S2. Percentage of $(Li_2S)_x(P_2S_5)_{1-x}$ structures that can be uniquely assigned to a reference crystal structure based on their Pearson similarity value S_p. The y axis, the *Similarity Margin*, is the similarity difference to the second most similar reference structure. Data for four different similarity thresholds are shown. In the present work, we considered only structures that have a similarity of $S_p \geq 0.4$ with any of the reference structures (green curve). With this choice, more than 95% of the structures can be assigned uniquely to a reference structure subject to a similarity margin of 0.2.

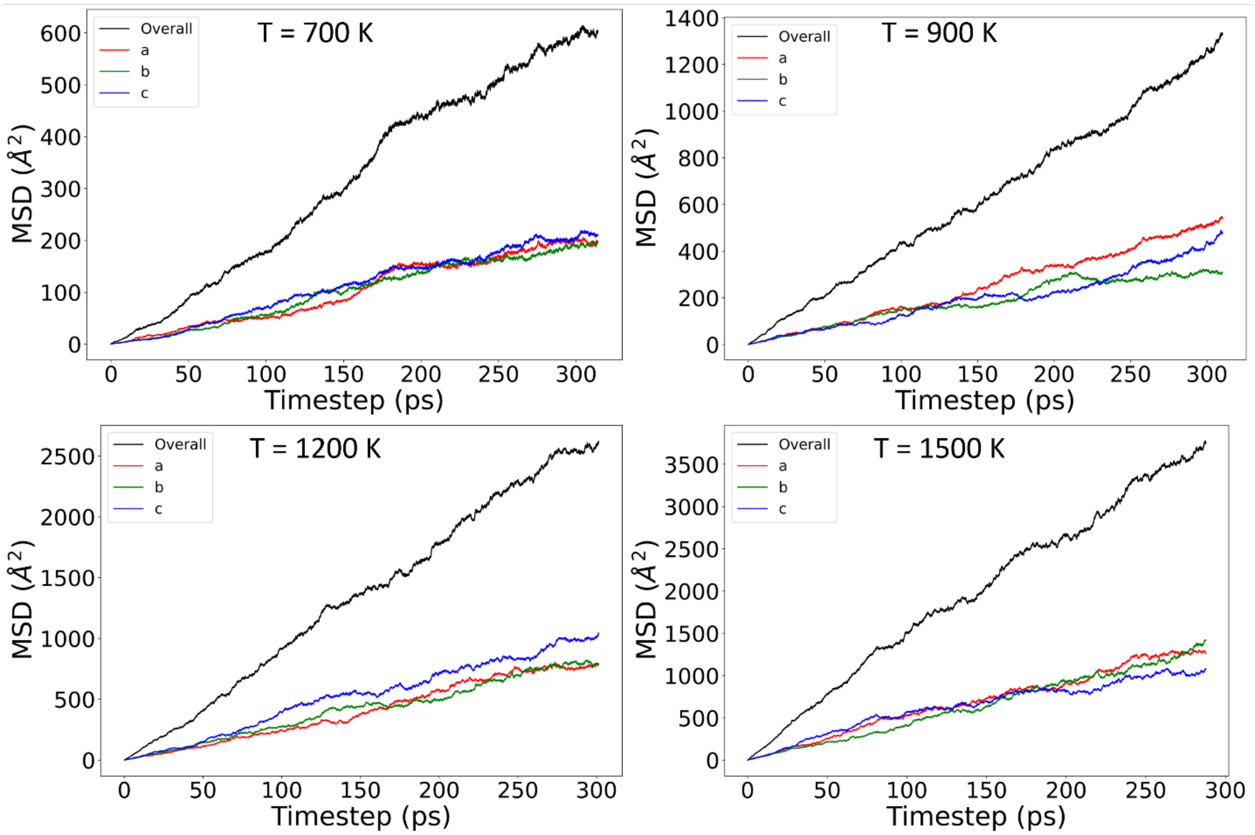

Figure S3. The mean-squared-displacement (MSD) of Li ions in *gc*-$Li_{42}P_{16}S_{61}$ at elevated temperatures (700 K, 900 K, 1200 K and 1500 K).

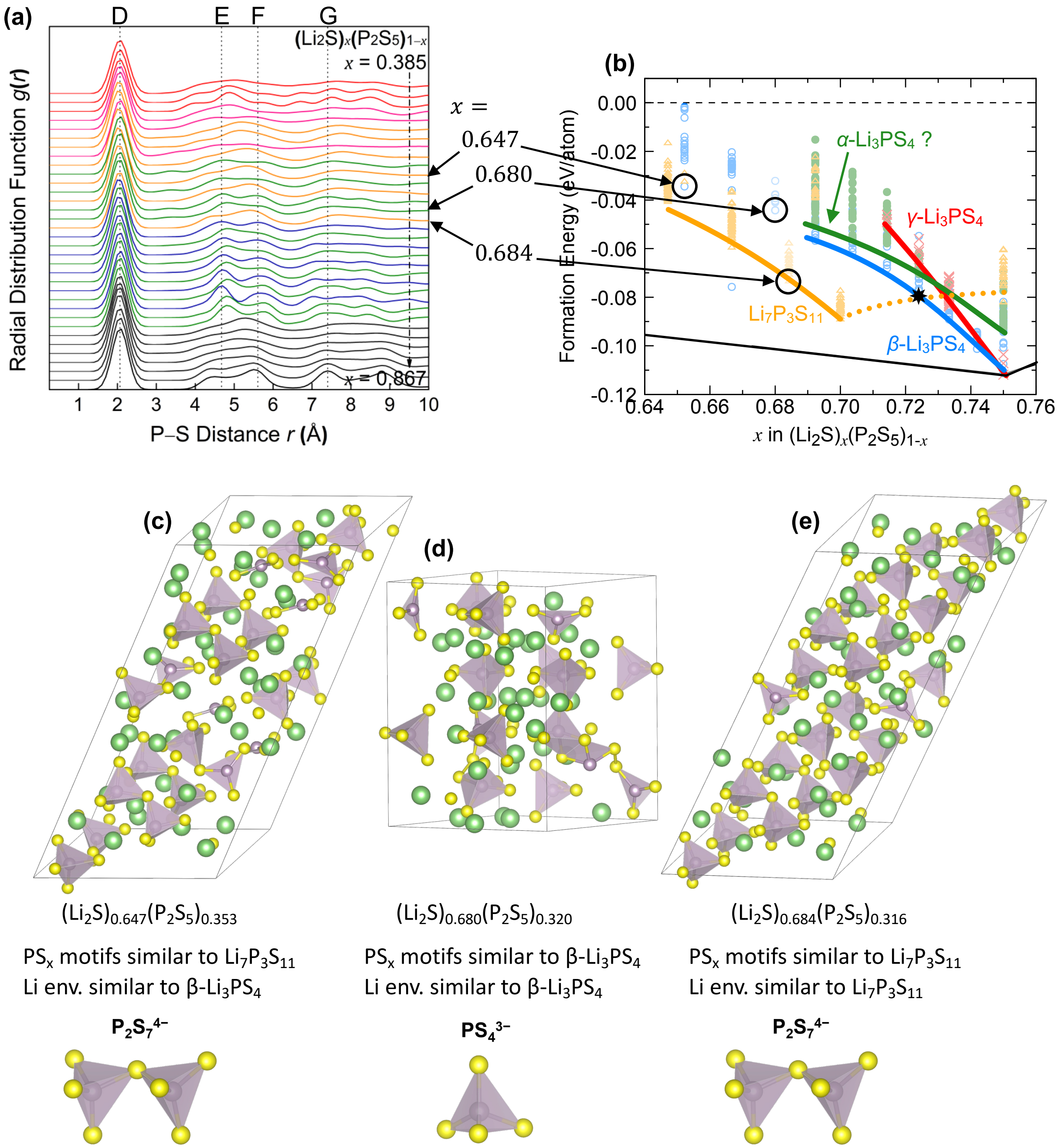

Figure S4. **(a)** P–S radial distribution functions (RDFs), **(b)** cluster analysis of Li site environments, **(c)-(d)** visualization and analysis of exemplary sampled structures. As also established in prior work, the P–S RDF shown in panel (a) correlates with the PS_x motifs present in a structure. The cluster analysis of panel (b) allows the comparison of Li site environments. In the sampled structures, the Li environment is not necessarily defined by the PS_x motifs. One example is the $(Li_2S)_{0.647}(P_2S_5)_{0.353}$ structure shown in panel (c), which exhibits $P_2S_7^{-4}$ motifs as its parent crystal structure $Li_7P_3S_{11}$, whereas the Li environments are more similar to those in the β-Li_3PS_4 crystal structure. In the other two examples shown in panels (d) and (e), the PS_x motifs and the Li environment are inherited from the parent structure.

REFERENCES

(1) Onodera, Y.; Mori, K.; Otomo, T.; Sugiyama, M.; Fukunaga, T. Structural Evidence for High Ionic Conductivity of Li7P3S11 Metastable Crystal. *J. Phys. Soc. Jpn.* **2012**, *81* (4), 044802. https://doi.org/10.1143/JPSJ.81.044802.

(2) Ohara, K.; Mitsui, A.; Mori, M.; Onodera, Y.; Shiotani, S.; Koyama, Y.; Orikasa, Y.; Murakami, M.; Shimoda, K.; Mori, K.; Fukunaga, T.; Arai, H.; Uchimoto, Y.; Ogumi, Z. Structural and Electronic Features of Binary Li2S-P2S5 Glasses. *Sci. Rep.* **2016**, *6* (1), 21302. https://doi.org/10.1038/srep21302.

(3) Dietrich, C.; Sadowski, M.; Sicolo, S.; Weber, D. A.; Sedlmaier, S. J.; Weldert, K. S.; Indris, S.; Albe, K.; Janek, J.; Zeier, W. G. Local Structural Investigations, Defect Formation, and Ionic Conductivity of the Lithium Ionic Conductor Li4P2S6. *Chem. Mater.* **2016**, *28* (23), 8764–8773. https://doi.org/10.1021/acs.chemmater.6b04175.

(4) Dietrich, C.; Weber, D. A.; Culver, S.; Senyshyn, A.; Sedlmaier, S. J.; Indris, S.; Janek, J.; Zeier, W. G. Synthesis, Structural Characterization, and Lithium Ion Conductivity of the Lithium Thiophosphate Li2P2S6. *Inorg. Chem.* **2017**, *56* (11), 6681–6687. https://doi.org/10.1021/acs.inorgchem.7b00751.

(5) Dietrich, C.; Weber, D. A.; Sedlmaier, S. J.; Indris, S.; Culver, S. P.; Walter, D.; Janek, J.; Zeier, W. G. Lithium Ion Conductivity in Li2S–P2S5 Glasses – Building Units and Local Structure Evolution During the Crystallization of Superionic Conductors Li3PS4, Li7P3S11 and Li4P2S. *J. Mater. Chem. A* **2017**, *5* (34), 18111–18119. https://doi.org/10.1039/C7TA06067J.

(6) Smith, J. G.; Siegel, D. J. Low-Temperature Paddlewheel Effect in Glassy Solid Electrolytes. *Nat. Commun.* **2020**, *11* (1), 1483. https://doi.org/10.1038/s41467-020-15245-5.

(7) Garcia-Mendez, R.; Smith, J. G.; Neuefeind, J. C.; Siegel, D. J.; Sakamoto, J. Correlating Macro and Atomic Structure with Elastic Properties and Ionic Transport of Glassy Li2S-P2S5 (LPS) Solid Electrolyte for Solid-State Li Metal Batteries. *Adv. Energy Mater.* **2020**, *10* (19), 2000335. https://doi.org/10.1002/aenm.202000335.

(8) Self, E. C.; Chien, P.-H.; O'Donnell, L. F.; Morales, D.; Liu, J.; Brahmbhatt, T.; Greenbaum, S.; Nanda, J. Investigation of Glass-Ceramic Lithium Thiophosphate Solid Electrolytes Using NMR and Neutron Scattering. *Mater. Today Phys.* **2021**, 100478. https://doi.org/10.1016/j.mtphys.2021.100478.
